# Learning with privileged and sensitive information: a gradient-boosting approach

**DOI:** 10.3389/frai.2023.1260583

**Published:** 2023-11-13

**Authors:** Siwen Yan, Phillip Odom, Rahul Pasunuri, Kristian Kersting, Sriraam Natarajan

**Affiliations:** ^1^Computer Science Department, University of Texas at Dallas, Dallas, TX, United States; ^2^Georgia Tech Research Institute, Georgia Institute of Technology, Atlanta, GA, United States; ^3^Amazon, Seattle, WA, United States; ^4^Department of Computer Science, Hessian Center for AI (hessian.AI), Technical University of Darmstadt, Darmstadt, Germany

**Keywords:** privileged information, fairness, gradient boosting, knowledge-based learning, sensitive features

## Abstract

We consider the problem of learning with sensitive features under the privileged information setting where the goal is to learn a classifier that uses features not available (or too sensitive to collect) at test/deployment time to learn a better model at training time. We focus on tree-based learners, specifically gradient-boosted decision trees for learning with privileged information. Our methods use privileged features as knowledge to guide the algorithm when learning from fully observed (usable) features. We derive the theory, empirically validate the effectiveness of our algorithms, and verify them on standard fairness metrics.

## 1. Introduction

Machine learning methods that consider learning from sources beyond just a single set of labeled data have long been explored under several paradigms—learning with advice (Towell and Shavlik, 1994; Fung et al., 2002; Maclin et al., 2005; Kunapuli et al., 2013; Das et al., 2021), learning from preferences (Boutilier, 2002; Drummond and Boutilier, 2014; Pang et al., 2018), learning from qualitative constraints (Altendorf et al., 2005; Yang et al., 2013; Kokel et al., 2020), active learning (Settles, 2012), transductive learning (Joachims, 1999), and as knowledge injection inside deep learning (Ding et al., 2018; Wang and Pan, 2020; Bu and Cho, 2021).

We view the problem of learning with sensitive information using the lens of privileged information. For instance, in a clinical study for improving adverse pregnancy outcomes, it is natural to solicit information about race or sexual orientation. While race can potentially affect the prior chances of an outcome (e.g., gestational diabetes or pre-term birth), the treatment plan in the clinic should not discriminate based on this feature. Similarly, while age/zipcode could be important to obtain a prior about the capacity to repay a loan, it should not be used as a feature (due to its sensitive nature) during deployment of the system. Chouldechova et al. (2018) consider these sensitive information as non-discriminatory for fair machine learning. Kilbertus et al. (2018) encrypt sensitive attributes, and Williamson and Menon (2019) measure the fairness risk on sensitive features.

In a different direction, Vapnik and Vashist (2009) introduced the problem of learning from privileged information where more information in the form of features is provided during training but is not available during testing/deployment. These *privileged features* could be sensitive features (race/age/sexual orientation) or features that are simply too expensive to collect during deployment (expensive sensors or FMRIs—functional magnetic resonance imaging, in a small clinic). Hence, the classifier cannot use these privileged features during deployment but may still be able to use them to improve the quality of the model.

Our key idea is to use the privileged features as an “inductive bias” or as knowledge constraints. To this effect, we develop two versions of the gradient boosting algorithm—in the first approach, a prior model is learned on the privileged/sensitive features. This prior model is then used to constrain the model learned from the fully observable features. Since this is inspired from the knowledge-based learning literature, we refer to this as **KbPIB** (*knowledge-based privileged information boosting*). In the second approach, the models over the privileged/sensitive features and the observed features are learned in a joint stage-wise manner. At each iteration, first a small tree is learned on the privileged features, the set of which is used as constraints for the observed feature model and the process is repeated. Since these are learned jointly, we refer to this as **JPIB** (*joint privileged information boosting*). The intuition is that while the privileged features provide extra information, they are not fully relied on when building the model. The resulting model, in essence, is a trade-off between the privileged information and the fully observed features—as is typically done in advice-based methods where the data and the expert knowledge are explicitly considered when learning.

We make a few key contributions: First, inspired by Quadrianto and Sharmanska (2017), we pose the problem of fair machine learning with sensitive data using the framework of privileged information. Second, we present algorithms for learning trees *via* functional-gradient boosting and show the gradient updates. Specifically, we derive two different types of boosted algorithms that can effectively exploit the sensitive/privileged features. Finally, we perform exhaustive empirical evaluation that demonstrates the effectiveness of the proposed approaches on different types of test beds—standard benchmark data sets, fairness data sets with sensitive information, and real-world medical data sets where the goals are to predict gestational diabetes, nephrotic syndrome, and rare disease occurrences. The results across data sets and evaluation metrics (including fairness metrics) clearly show the effectiveness of the algorithms.

## 2. Background

### 2.1. Learning with privileged information

Learning with privileged information is inspired by richer forms of interactions between human teachers and students (Vapnik and Vashist, 2009). Particular (labeled) examples are given to the student along with explanations and intuitions that are able to speed up the comprehension of novel concepts. More formally, learning with privileged information assumes that more information is known about the training examples. However, as the expert is not available for testing, this additional information is not available at test time. Thus, training examples have the form $\langle y_{i},{\text{x}}_{i}^{\text{C}\text{F}},{\text{x}}_{i}^{\text{P}\text{F}}\rangle$ while testing examples have the form $\langle y_{i},{\text{x}}_{i}^{\text{C}\text{F}}\rangle$. **CF** refers to the classifier/normal features available during testing and **PF** refers to the privileged features.

Learning algorithms for privileged information have previously focused on SVMs (Vapnik and Vashist, 2009; Sharmanska et al., 2013). The original formulation—SVM+ (Vapnik and Vashist, 2009)—learned the difficulty of each training example. The key idea was to learn an SVM in the privileged space (using $\left\{\langle y_{i},{\text{x}}_{i}^{\text{P}\text{F}}\rangle \right\}$) and find the margin with respect to this SVM for each training example. Training examples closer to the margin are considered “more difficult” as they are closer to the decision boundary while examples farther from the margin are considered “less difficult.” Since the introduction of the new learning paradigm and the corresponding SVM+ approach, there is a growing body of work on learning with privileged information. Pechyony and Vapnik (2010) developed a theoretical justification of the learning setting. Liang et al. (2009) established links between the SVM+ and the multi-task learning. Hernández-Lobato et al. (2014) showed that the privileged information can naturally be treated as noise in the latent function of a Gaussian process classifier (GPC). In contrast to the standard GPC setting, the latent function becomes a natural measure of confidence about the training data by modulating the slope of the GPC sigmoid likelihood function.

Most closely related to our study, Chen et al. (2012) extend the setting to AdaBoost, and Lapin et al. (2014) relate privileged information to importance weighting within SVMs. Decision tree learners, however, have not been explored in this context yet. Instead of giving more importance to certain examples, we establish a novel connection to knowledge-based machine learning that relies on existing knowledge (Section 3.2). We show that the knowledge we have beforehand, which can be described with privileged features, can also be represented using labels assigned to each training example. These labels help guide the learning process. Moreover, we improve the learning process by introducing a regularization term into the log-likelihood for boosting method. This regularization term is calculated as the KL divergence between the distribution using classifier features and the distribution using privileged features, which are available only at training time and not during testing.

While our setting is similar to the generalized distillation (Lopez-Paz et al., 2016), the fundamental principles are different. We focus on two sets of features—privileged features and normal features. Our goal is to build a model (teacher model) on privileged features that guides the learning of a model (student model) on normal features to improve performance. While our strategy at a high-level appears similar to knowledge distillation by Hinton et al. (2015), there are notable and important differences as follows: (1) the teacher function and student function are learned sequentially, and (2) predictions of teacher model are included as soft labels for the student model.

Our study is also related to knowledge injection in deep networks. Ding et al. (2018) use mean images as (color) knowledge to produce class weight and object occurrence frequencies as scene knowledge to determine scene weight; Wang and Pan (2020) integrate logical knowledge in the form of first-order logic as knowledge regularization into deep learning system; Bu and Cho (2021) perform neuro-symbolic integration using domain knowledge as first-order logic rules.

### 2.2. Functional gradient boosting

Many probabilistic learning methods learn the conditional distribution *P*(*y*_*i*_|**x**_*i*_; ψ) using standard techniques such as gradient-descent that is usually performed on the log-likelihood w.r.t. parameters to find the best set of parameters that model the training data. Functional Gradient Boosting methods (GB; Friedman, 2001; Dietterich et al., 2008; Natarajan et al., 2012, 2015), on the other hand, represent the likelihood in a functional form (typically using the sigmoid function) $P\left(y_{i}|{\text{x}}_{i};\psi \right)=\frac{e^{\psi \left(y_{i},{\text{x}}_{i}\right)}}{\underset{y^{\prime }}{\sum }e^{\psi \left(y^{\prime },{\text{x}}_{i}\right)}}$ where ψ is a regression function defined over the examples. Given this representation, GB methods obtain the gradient of the log-likelihood w.r.t. ψ(*y*_*i*_ = 1, **x**_*i*_) for each training example, *x*_*i*_ as: Δ(*y*_*i*_) = *I*(*y*_*i*_ = 1)−*P*(*y*_*i*_ = 1|**x**_*i*_; ψ), where *I* is an indicator function which returns 1 for positive examples and 0 for negative examples in a binary classification task. The GB approach starts with an initial regression function, ψ_0_ = 0 to compute the probabilities of the training examples and thereby the gradients Δ_1_. A regression function (typically a tree), ${\hat{\Delta }}_{1}$, is fit on the training examples with the gradients as the target regression values. This learned function is now added to ψ_0_, and the process is repeated with $\psi_{1}=\psi_{0}+{\hat{\Delta }}_{1}$. Given that the stage-wise growth of trees resembles boosting, and that the process involves computing gradients of functions, this method is called *Gradient Boosting* (GB).

### 2.3. Sensitive features in fairness

Several studies within fairness in ML treat the sensitive information (e.g., race, gender, or financial status) as non-discriminatory (Žliobaitė, 2017; Chouldechova et al., 2018). Several approaches have been proposed to avoid the use of sensitive features, including by utilizing encrypted sensitive attributes (Choudhuri et al., 2017; Kilbertus et al., 2018) or utilizing sensitive features to measure the fairness risk by proposing a new definition of fairness to include categorical or real-valued sensitive groups beyond binary sensitive features (Angwin et al., 2016; Williamson and Menon, 2019). Krasanakis et al. (2018) reweigh training samples on trade-offs between accuracy and disparate impact. Kamishima et al. (2012) regularize on prejudice (a statistical dependence between sensitive features and other information) to achieve fairness. Quadrianto and Sharmanska (2017) enforce fairness constraints through privileged learning. They consider the setting from the study by Vapnik and Vashist (2009) to build a privileged model on all features, optimizing the prediction boundary of a privileged model and adapting the boundary of the normal model. On the other hand, we use a boosted model as the privileged model relying only on the privileged features and incorporate constraints from the privileged model into the objective of the model constructed on non-privileged features. Wang et al. (2021) approach fairness by putting strict restraints on the ability to infer sensitive features from the available features. Their approach focuses on improving fairness while maintaining performance. Alternatively, our approach aims to leverage the sensitive information to improve performance. Empirically, we demonstrate that our approach maintains fairness.

## 3. Boosting with privileged sensitive information

**Motivating real-world task**: The Nulliparous Pregnancy Outcomes Study (NuMoM2b) monitors expectant mothers with the goal of predicting adverse pregnancy outcomes (Haas et al., 2015). The data set includes clinical tests (e.g., BMI, METs) and demographic information. Our goal is to use this data to predict gestational diabetes. While the prevalence of gestational diabetes varies significantly across ethnic groups, it may not be appropriate to use the sensitive demographic information to make the diagnoses. Thus, we may utilize this privileged information during training but want to withhold it from our diagnostic models. A similar consideration is in our rare disease data where certain demographic information, such as age, gender, and marital status, is considered privileged and cannot be used during deployment. While we focus on four specific medical tasks, one could imagine such situations in other high social impact problems including, but not limited to, credit card/home/auto/education loan approvals, hiring decisions, clinical study recruitment, or allocation of resources, where some sensitive information could be used while training to better understand the problem but cannot be used during deployment.

### 3.1. Problem formulation

Recalling that our goal is to learn robust models that do not include sensitive/privileged information but still leverage them to improve training. Our problem is formally defined as follows:

**Given:** A set of training examples $\left\{\langle y_{i},{\text{x}}_{i}^{\text{C}\text{F}},{\text{x}}_{i}^{\text{P}\text{F}}\rangle \right\}$ and a set of test examples $\left\{\langle y_{i},{\text{x}}_{i}^{\text{C}\text{F}}\rangle \right\}$, where

$$\begin{array}{l} \text{F}=\text{C}\text{F}\cup \text{P}\text{F}\;\&\;\text{C}\text{F}\cap \text{P}\text{F}=\emptyset \end{array}$$

**To Do:** Learn a classifier that employs only the classifier features **CF** for classifying the test data and can utilize the privileged features **PF** effectively in learning a better model.

**F** is the set of all features, **CF** is the set of features that are available at both training and testing time (and we call them *classifier features*), **PF** are the privileged features that are accessible only during training and not during testing, *y*_*i*_ is the label of the *ith* example and **x**_*i*_ is the feature of that example. We use {} to denote sets. For example, the input to the algorithm is the set of all examples $\left\{\langle y_{i},{\text{x}}_{i}^{\text{C}\text{F}},{\text{x}}_{i}^{\text{P}\text{F}}\rangle \right\}$. We first consider a knowledge-based approach to leverage with privileged features. Then, we extend this approach to joint training over the classifier and the privileged features. While we use tree-based classifiers, our approach can easily be extended to other clustering/classification techniques.

### 3.2. Knowledge-based privileged information boosting

**Figure 1:**
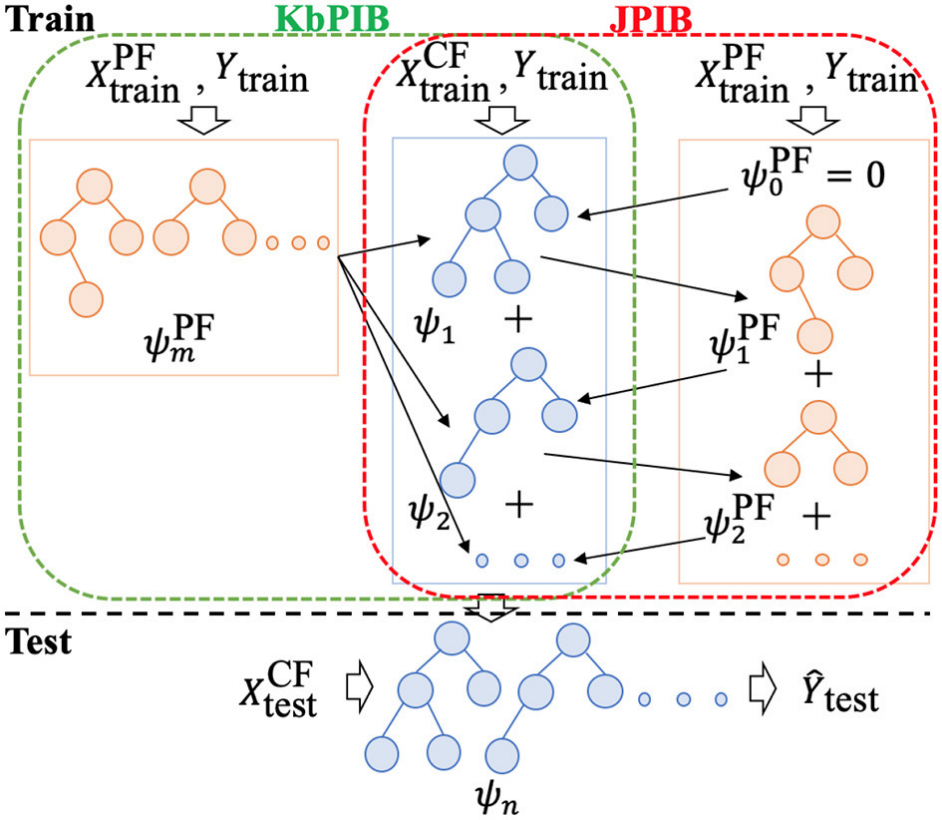
Overview of proposed approaches KbPIB and JPIB [Both approaches train a model on CF, while KbPIB trains a model on PF once and uses it as a bias for the classifier, JPIB learns models on CF and PF in an iterative manner (in a manner loosely similar to co-training); models on PF data are dropped after training to avoid consideration during testing/deployment].

Inspired by knowledge-based machine learning methods, Fung et al. (2002), for example, reformulated SVM classifier that uses previous knowledge in the form of multiple polyhedral sets; Kunapuli et al. (2013) incorporate expert advice in states and actions by stating preferences; Towell and Shavlik (1994) map domain theories in propositional logic into neural networks, which leverage external knowledge *via* human input to guide the learning process; we consider privileged information as a source of high-quality knowledge. We introduce two models: a model learned over the classifier features and a privileged model that is learned over the privileged features in Algorithm 1. By attempting to guide the predictions of the classifier model with the privileged model, we can potentially find a way to insert informed priors to the labels based on both the privileged and classifier features. We first train a model over privileged features at line 2 in Algorithm 1. Then, from lines 3 to 9, we learn a model over the classifier features while reducing the margin with the privileged model. An overview of our **KbPIB** approach is shown in Figure 1.

**Algorithm 1:** **KbPIB**: Knowledge-based Privileged Information Boosting.

**Input**: Classifier features: training data $X_{\text{train}}^{\text{C}\text{F}},Y_{\text{train}}$; validation data $X_{\text{val}}^{\text{C}\text{F}},Y_{\text{val}}$; privileged features: training data $X_{\text{train}}^{\text{P}\text{F}},Y_{\text{train}}$; validation data $X_{\text{val}}^{\text{P}\text{F}},Y_{\text{val}}$
**Parameter**: Number of trees *N*, early-stop parameter *P***Output**: Learned model ψ
1: Initialize model ψ_0_ = 0, counter *C* = 0, score *R*, best number of trees index *j*
2: ψ^**PF**^← **NF**($X_{\text{train}}^{\text{P}\text{F}},Y_{\text{train}},X_{\text{val}}^{\text{P}\text{F}},Y_{\text{val}}$) { Supplementary Algorithm 1}
3: **for** *i* = 1 **to** *N* **do**
4: Δ_*i*_← ComputeGradient($X_{\text{train}}^{\text{C}\text{F}},Y_{\text{train}},\psi_{i-1},\psi^{\text{P}\text{F}}$) {Equation (2)}
5: ${\hat{\Delta }}_{i}\leftarrow$ FitRegressionValue($X_{\text{train}}^{\text{C}\text{F}},\Delta_{i}$)
6: $\psi_{i}\leftarrow \psi_{i-1}+{\hat{\Delta }}_{i}$
7: *R*_val_ ← Evaluate($X_{\text{val}}^{\text{C}\text{F}},Y_{\text{val}},\psi_{i}$)
8: *j*, *R*, *C* ← EarlyStop(*i*, *j*, *R*, *R*_val_, *C*, *P*) { Supplementary Algorithm 2}
9: **end for**
10: **return** ψ_*j*_

In Vapnik's SVM+ model, the **PF** features were used to define an oracle function that can predict the slack on each example. In our probabilistic framework, we use the **PF** features to build an oracle model that can predict a close approximation to the true distribution of each example which is not captured by the discrete class labels. Instead of modeling the error (distance between the labels and the underlying distribution), we directly model the distribution of labels using privileged features during training. We use *P*(*y*|**x**^**PF**^; ψ′) to indicate this true label distribution learned over privileged features and *P*(*y*|**x**^**CF**^; ψ) for the distribution learned over classifier features. Similar to SVM+, we can now use the privileged features to model this difference between the true distribution and the label distribution. Since the training labels are completely observed, the privileged features can be directly used to model the distribution of the examples. Thus, we learn a model that minimizes the error of the model over the training labels and the margin between the distribution *P*(*y*|**x**^**PF**^; ψ′) and *P*(*y*|**x**^**CF**^; ψ),


$$\min_{\psi }\sum_{i}(\underset{\text{NLL}}{\underset{︸}{-\log P(y_{i}|x_{i}^{CF};\psi )}}+\alpha \cdot \underset{\text{KLDivergence}}{\underset{︸}{\text{KL}(P(y_{i}|x_{i}^{PF};\psi^{\prime })||P(y_{i}|x_{i}^{CF};\psi ))}})$$

NLL denotes the negative log-likelihood of the training data that models the error while KL denotes the KL divergence between *P*(*;ψ′) and *P*(*;ψ) and is equal to $\underset{i}{\sum }P\left(i;\psi^{\prime }\right)\log\frac{P\left(i;\psi^{\prime }\right)}{P\left(i;\psi \right)}$. We use α to model the trade-off between fitting to the labeled data versus fitting to the distribution learned over the privileged features. We can now use gradient boosting with respect to $\psi \left(y_{i}=1,{\text{x}}_{i}^{\text{C}\text{F}}\right)$ to minimize this objective function.

Notably, in our formulation, the model ψ′ could be provided by the domain expert on the privileged features (for instance, a Bayesian network or a neural network that is used in the literature on these privileged features). We do not assume any specific form for ψ′, and the goal is to use this privileged knowledge. In our experiments, we learn ψ′ from data. If the model of ψ′ is provided, one could treat that as a regularizer (similar to knowledge-based learning).

The first term of our objective function is the standard log-likelihood function which has the gradient as follows^1^:


(1) $$\begin{array}{l} \frac{\partial \underset{i}{\sum }\log P\left(y_{i}|{\text{x}}_{i}^{\text{C}\text{F}};\psi \right)}{\partial \psi \left(y_{j}=1,{\text{x}}_{j}^{\text{C}\text{F}}\right)}=I\left(y_{j}=1\right)-P\left(y_{j}=1|{\text{x}}_{j}^{\text{C}\text{F}};\psi \right) \end{array}$$

For the second term, we derive the gradients below:


$$\begin{array}{l} \frac{\partial \text{KL}(P(y_{i}|x_{i}^{PF};\psi^{\prime })||P(y_{i}|x_{i}^{CF};\psi ))}{\partial \psi (y_{i}=1,x_{i}^{CF})}\\ =\frac{\partial \sum_{y_{i}}P(y_{i}|x_{i}^{PF};\psi^{\prime })\left(\log P(y_{i}|x_{i}^{PF};\psi^{\prime })-\log P(y_{i}|x_{i}^{CF};\psi )\right)}{\partial \psi (y_{i}=1,x_{i}^{CF})}\\ =-\frac{\partial \sum_{y_{i}}P(y_{i}|x_{i}^{PF};\psi^{\prime })\log P(y_{i}|x_{i}^{CF};\psi )}{\partial \psi (y_{i}=1,x_{i}^{CF})}\\ =-(P(y_{i}=1|x_{i}^{PF};\psi^{\prime })\frac{\partial \log P(y_{i}=1|x_{i}^{CF};\psi )}{\partial \psi (y_{i}=1,x_{i}^{CF})}\\ +P(y_{i}=0|x_{i}^{PF};\psi^{\prime })\frac{\partial \log P(y_{i}=0|x_{i}^{CF};\psi )}{\partial \psi (y_{i}=1,x_{i}^{CF})})\\ =-(P(y_{i}=1|x_{i}^{PF};\psi^{\prime })\cdot (1-P(y_{i}=1|x_{i}^{CF};\psi ))\\ +P(y_{i}=0|x_{i}^{PF};\psi^{\prime })\cdot (-P(y_{i}=1|x_{i}^{CF};\psi )))\\ =P(y_{i}=1|x_{i}^{CF};\psi )-P(y_{i}=1|x_{i}^{PF};\psi^{\prime }) \end{array}$$

We combine the gradient terms to get the final gradient for each example as follows^2^:


(2) $$\begin{array}{l} \Delta (x_{i}^{CF})=I(y_{i}=1)-P(y_{i}=1|x_{i}^{CF};\psi )\\ \;-\alpha \cdot \left(P(y_{i}=1|x_{i}^{CF};\psi )-P(y_{i}=1|x_{i}^{PF};\psi^{\prime })\right) \end{array}$$

Intuitively, if the learned distribution has a higher probability of an example belonging to the positive class compared with the distribution, $P\left(y_{i}=1|{\text{x}}_{i}^{\text{C}\text{F}};\psi \right)-P\left(y_{i}=1|{\text{x}}_{i}^{\text{P}\text{F}};\psi^{\prime }\right)$ would be positive and the gradient would be pushed lower. Hence, the additional term would push the gradient (weighted by α) toward the distribution as predicted by our privileged features.

The parameter α controls the influence of the privileged data on the learned distribution. When α = 0, privileged features are ignored resulting in the standard functional gradient. As α is increased, the gradient is pushed lower, for example, where predicted probability is higher than true probability (w.r.t. privileged model) and vice versa.

### 3.3. Joint privileged information boosting

While the previous approach used the privileged information to influence final model learned over **CF** at each step in gradient boosting, it did not leverage this learned model to further tune the privileged tree labels. By attempting to reduce the margin by jointly training the two models, we can potentially find more consistent predictions based on both the privileged and classifier features. Similar to Equation (2), the gradients can be computed for learning the true distribution using the privileged features with *P*(*;ψ) and *P*(*;ψ′) switched around.


(3) $$\begin{array}{l} \Delta (x_{i}^{PF})=[I(y_{i}=1)-P(y_{i}=1|x_{i}^{PF};\psi^{\prime })]\\ \;-\alpha \cdot [\left(P(y_{i}=1|x_{i}^{PF};\psi^{\prime })-P(y_{i}=1|x_{i}^{CF};\psi )\right)] \end{array}$$

Given these gradients, we can now describe our approach called **JPIB** to perform gradient boosting jointly over the classifier features and the privileged information. We iteratively learn regression functions (trees in our case) to fit to these gradients. However, the key difference from **KbPIB** is that we perform co-ordinate gradient descent, i.e., we alternate between taking a gradient step along ψ and ψ′. From lines 2 to 11 in Algorithm 2, we learn one regression tree using the gradients based on the classifier features (lines 2–5), compute the gradients for the privileged features, learn a tree for the privileged features (lines 8–10), and repeat this at most *N* times to generate at most *N* trees of the boosting model. The early-stop mechanism at line 7 helps return the best performing model on validation data (line 6).

**Algorithm 2:** **JPIB**: Joint Privileged Information Boosting.

**Input**: Classifier features: training data $X_{\text{train}}^{\text{C}\text{F}},Y_{\text{train}}$, validation data $X_{\text{val}}^{\text{C}\text{F}},Y_{\text{val}}$; privileged features: training data $X_{\text{train}}^{\text{P}\text{F}},Y_{\text{train}}$
**Parameter**: Number of trees *N*, early-stop patience *P***Output**: Learned model ψ
1: Initialize models $\psi_{0}^{\text{P}\text{F}}=0$ and ψ_0_ = 0, counter *C* = 0, score *R*, best number of trees index *j*
2: **for** *i* = 1 **to** *N* **do**
3: Δ_*i*_← ComputeGradient($X_{\text{train}}^{\text{C}\text{F}},Y_{\text{train}},\psi_{i-1},\psi_{i-1}^{\text{P}\text{F}}$) {Equation (2)}
4: ${\hat{\Delta }}_{i}\leftarrow$ FitRegressionValue($X_{\text{train}}^{\text{C}\text{F}},\Delta_{i}$)
5: $\psi_{i}\leftarrow \psi_{i-1}+{\hat{\Delta }}_{i}$
6: *R*_val_ ← Evaluate($X_{\text{val}}^{\text{C}\text{F}},Y_{\text{val}},\psi_{i}$)
7: *j*, *R*, *C* ← EarlyStop(*i*, *j*, *R*, *R*_val_, *C*, *P*) {Supplementary Algorithm 2}
8: $\Delta_{i}^{\text{P}\text{F}}\leftarrow$ ComputeGradient($X_{\text{train}}^{\text{P}\text{F}},Y_{\text{train}},\psi_{i-1}^{\text{P}\text{F}},\psi_{i}$) {Equation (3)}
9: ${\hat{\Delta }}_{i}^{\text{P}\text{F}}\leftarrow$ FitRegressionValue($X_{\text{train}}^{\text{P}\text{F}},\Delta_{i}^{\text{P}\text{F}}$)
10: $\psi_{i}^{\text{P}\text{F}}\leftarrow \psi_{i-1}^{\text{P}\text{F}}+{\hat{\Delta }}_{i}^{\text{P}\text{F}}$
11: **end for**
12: **return** ψ_*j*_

### 3.4. Sensitive attributes and fairness constraints

Notably, since our algorithms drop the privileged information after learning, one could argue that they do not discriminate between the different groups at deployment time. However, one could go even deeper and establish **a strong connection between the learning framework and the fairness constraints**. Given the above definitions of the objective function, several fairness constraints can be easily captured by our model. For instance, to handle **metric fairness**, the privileged model could simply be a constraint of the form


(∀x,y)sim(x,y)⇒h(x)=h(y)

that can be used inside the second term of Equations (2) and (3), where the second term is the probability of the constraint satisfied by the model (computed by counting). **Weakly meritocratic fairness** can be handled by the form


(∀x,y)merit(x)≥merit(y)⇒ψ(x)>ψ(y)

while **group fairness** can be handled by


normalgroup(x)∧protectgroup(y)⇒h(x)=h(y)

and **group parity** can be handled by using precision and recall. Similar to the metric constraints, all these constraints can be included in the second term of the model. Essentially one could drop the privileged tree model and use these constraints. Another way is to include these constraints along with the privileged model. However, as we show in our experiments, with treating the sensitive attributes as privileged features, the algorithm performs significantly better in terms of the fairness criteria compared with the boosting baseline.

## 4. Experiments

Our experimental evaluations aim to answer the following questions:

**Q1:** How effective is incorporating privileged information into gradient boosting?
**Q2:** Can jointly updating the privileged model with the classifier improve performance?
**Q3:** How is model fairness affected by withholding sensitive information from the classifier?

We present empirical evaluations of our proposed approaches—(**KbPIB**) and (**JPIB**). We evaluate the approaches in two ways. To evaluate the effect of privileged information, we compare against learning a gradient-boosted model over only the classifier/normal features, **NF**. To evaluate fairness, our approaches are compared with **All**, which is learned over both **CF** and imputed **PF** based on mode. Notably, though we explicitly evaluate against the SVM-based approach and fairness approach, the key question in our study is whether the notion of privileged information can help gradient boosting and whether the sensitive features are handled appropriately. We adopt 10-fold cross-validation for all datasets: 8-folds of training, 1-fold of validation, and 1-fold of test. Due to the data size and very few negative instances in the dataset Nephrotic Syndrome, we use 5-fold cross-validation: 3-folds of training, 1-fold of validation, and 1-fold of test. The value of α and thresholds of precision and recall are selected based on the validation data. More details are presented in Supplementary material. The experiments are conducted on the machine with CentOS Linux 7, CPU of Intel Xeon E5-2630 with 2.40 GHz and 16 cores, and 512 GB RAM. The source code (details of dependency) of our methods and prepared data can be downloaded.^3^

### 4.1. Datasets

We employ three types of datasets: standard benchmarks, medical datasets, and fairness benchmarks. The standard benchmarks consist of three datasets from UCI ML repository (Dheeru and Taniskidou, 2017). The fairness benchmarks include 12 datasets from 10 data sources: Adult (Kohavi, 1996), Diabetes (Diab.) (Strack et al., 2014), Dutch Census (Dutch) (Van der Laan, 2000), Bank Marketing (Bank) (Moro et al., 2014), Credit Card Clients (Credit) (Yeh and Lien, 2009), COMPAS (COMP.) and COMPAS Violence (C. V.) (Angwin et al., 2016), Student–Mathematics (St. M.) and Student–Portuguese (St. P.) (Cortez and Silva, 2008), OULAD (OUL.) (Kuzilek et al., 2017), Communities and Crime (Comm.), and KDD Census Income (KDD) (Dheeru and Taniskidou, 2017). While we describe the medical datasets in more detail, the properties of standard and fairness benchmarks are presented in Table 1.

**Table 1:** Standard benchmark datasets and fairness benchmark datasets.

**Dataset**	**PF**	**#F**	**#Instances**	**N/P**
Heart	Tests	13	297	1.17
Car	Main.	6	1,728	2.34
Spam	Word freq.	57	4,601	1.54
Adult	Age, race, sex	13	30,162	3.02
Diab.	Sex	17	46,176	3.13
Dutch	Sex	11	60,420	1.10
Bank	Age, mar.	16	45,211	7.55
Credit	Edu., mar., sex	23	30,000	3.52
COMP.	Race, sex	8	6,172	1.20
C. V.	Race, sex	8	4,015	5.16
Comm.	Race	21	1,994	15.34
St. M.	Age, sex	32	395	0.49
St. P.	Age, sex	32	649	0.18
OUL.	Sex	10	21,562	0.47
KDD	Race, sex	23	284,556	15.35

**PF**, privileged features; #**F**, #features; N/P, negative positive ratio; main., maintenance; edu., education; mar., marital status.

### 4.2. Real-world medical datasets

#### 4.2.1. NuMoM2b_a

Polygenic risk scores (PRS) for type 2 diabetes (T2D) can improve risk prediction for gestational diabetes (GD) (Haas et al., 2015). We use PRS as the privileged feature. Demographic information and clinical history serve as normal features: body mass index (BMI), exercise levels or metabolic equivalents of time (METs), age, diabetes history (DM_Hist), polycystic ovary syndrome (PCOS), and high blood pressure (HiBP). The classification task is to predict GD. There are 3,657 instances with Neg/Pos ratio of 25.89.

#### 4.2.2. NuMoM2b_b

We use the attribute race as privileged feature, which often is not usable during test or deployment for privacy concern (Haas et al., 2015). The normal features and classification task are same as NuMoM2b_a. There are 6,164 instances with the Neg/Pos ratio of 23.76.

#### 4.2.3. Nephrotic syndrome

A novel dataset of symptoms that indicates kidney damage is sourced from Dr Lal PathLabs, India.^4^ This consists of 50 clinical reports with patient history information. The privileged features are age and gender. History of other diseases, Edema duration, urine test, and blood reports are used as normal features. The classification task is to predict Nephrotic Syndrome. The Neg/Pos ratio is 0.14.

#### 4.2.4. Rare disease

This dataset is collected to identify rare diseases from behavioral data (MacLeod et al., 2016). We consider age, gender, and marital status as privileged features. The survey questions are used as normal features and include demographic information, disease information, technology use, and health care professional inputs. The boolean classification task is to predict the presence of rare diseases. There are 284 instances with the Neg/Pos ratio of 2.69 and 69 features.

### 4.3. Results

We first compare our **KbPIB** and **JPIB** approaches to the baseline **NF** that does not use privileged information during training. We evaluate the approaches based on the AUC ROC, as shown in Table 2, due to class imbalance. Blue denotes when either of our approaches outperform the baseline. The best performance is bolded. Overall, our approaches outperform the baseline, showing improvement in 18 out of 19 datasets. Both of our methods perform at least as well as the baseline across the rest of the datasets. Notably, both **KbPIB** (2 out of 4) and **JPIB** (3 out of 4) outperform the baseline in real-world medical tasks, where sensitive information includes demographic information. The NS dataset, on the other hand, has a large number of positives to negatives (but a small number of examples over all), and the base model that uses the urine tests gets nearly perfect example. It is an example of a situation where privileged information does not quite helpful, and it is natural that in many domains, the data might be sufficient to learn a good predictive model and extra information may not be helpful. We present this result to show the absence of improvement and acknowledge this case. **KbPIB** performs slightly worse than the baseline NF on three domains. In future, we can attempt different classifiers on privileged features and normal features.

**Table 2:** AUC ROC.

**Dataset**	**NF**	**KbPIB**	**JPIB**	**SVM+**
Heart	0.792	**0.810**	0.798	0.746
Car	0.845	**0.846**	**0.846**	0.841
Spam	0.961	0.961	**0.962**	0.934
N2b_a	0.658	0.656	0.684	**0.690**
N2b_b	0.643	0.652	**0.655**	0.641
NS	0.989	0.989	0.989	0.5
Rare	0.531	0.614	0.560	**0.667**
Adult	0.714	**0.725**	0.719	–
Diab.	0.562	0.561	**0.566**	–
Dutch	0.744	0.763	**0.764**	–
Bank	0.681	0.696	**0.714**	–
Credit	0.701	**0.703**	**0.703**	–
COMP.	0.618	0.627	0.643	**0.698**
C. V.	0.567	0.596	0.609	**0.703**
Comm.	0.893	0.883	0.899	**0.919**
St. M.	0.959	0.974	**0.975**	0.959
St. P.	0.908	**0.921**	0.914	0.914
OUL.	0.523	0.532	0.523	**0.534**
KDD	0.889	**0.890**	**0.890**	–

**KbPIB** and **JPIB** outperform the baseline **NF** in nearly all the datasets. Results with standard deviation in Supplementary material. “–” indicates out-of-memory error. Bold values are the best scores across different methods.

We also evaluate the approaches based on precision and recall in Table 3 due to class imbalance. In 6 out of 19 datasets, our approaches yield both higher precision and recall. Our approaches achieve higher precision and higher recall in 12 datasets. Collectively, our approaches that incorporate privileged information are able to achieve better performance across several metrics (**Q1**).

**Table 3:** Precision and recall in first and second rows, respectively.

**Dataset**	**NF**	**KbPIB**	**JPIB**
Heart	0.682 ± 0.0809	0.714 ±0.0819	0.761 ± 0.0774
	0.786 ± 0.1269	0.816 ±0.1117	0.707 ± 0.1300
Car	0.588 ± 0.0389	0.592 ±0.0384	0.581 ± 0.0342
	0.908 ± 0.0706	0.898 ±0.0800	**0.923** ± 0.0758
Spam	0.858 ± 0.0200	0.873 ±0.0218	0.859 ± 0.0248
	0.883 ± 0.0251	0.868 ±0.0270	0.884 ± 0.0268
N2b_a	**0.101** ± 0.0611	0.078 ±0.0476	0.093 ± 0.0645
	0.553 ± 0.3914	0.639 ± 0.3821	0.597 ± 0.4258
N2b_b	**0.081** ± 0.0548	0.065 ± 0.0503	0.064 ± 0.0379
	0.572 ± 0.3901	0.628 ± 0.4250	0.796 ± 0.3610
NS	0.960 ± 0.0894	0.960 ± 0.0894	0.960 ± 0.0894
	0.978 ± 0.0497	0.978 ± 0.0497	0.978 ± 0.0497
Rare	0.286 ± 0.0651	0.340 ± 0.0885	0.324 ± 0.1226
	**0.879** ± 0.2174	0.661 ± 0.1912	0.616 ± 0.2556
Adult	0.447 ± 0.0225	0.418 ± 0.0565	0.452 ± 0.0371
	0.631 ± 0.0229	0.704 ±0.1212	0.612 ± 0.0442
Diab.	0.243 ± 0.0029	0.245 ±0.0074	0.247 ± 0.0081
	**0.972** ± 0.0611	0.946 ± 0.1307	0.943 ± 0.0864
Dutch	**0.835** ± 0.0656	0.736 ± 0.1237	0.770 ± 0.1173
	0.572 ± 0.0662	0.682 ± 0.1439	0.663 ± 0.1423
Bank	0.308 ± 0.0300	0.304 ± 0.0292	0.312 ± 0.0483
	0.495 ± 0.1720	0.572 ± 0.1140	0.522 ± 0.1141
Credit	0.439 ± 0.0731	0.487 ± 0.1071	0.463 ± 0.0852
	**0.599** ± 0.0951	0.539 ± 0.1207	0.557 ± 0.1323
COMP.	0.520 ± 0.0476	0.555 ± 0.0798	0.559 ± 0.0698
	**0.825** ± 0.1840	0.666 ± 0.2403	0.676 ± 0.2124
C. V.	0.271 ± 0.0426	0.309 ± 0.0426	0.228 ± 0.0753
	0.340 ± 0.1536	0.434 ± 0.1490	0.584 ± 0.3006
Comm.	**0.505** ± 0.1356	0.447 ± 0.0957	0.485 ±0.1200
	0.411 ± 0.1249	0.515 ± 0.1398	0.485 ± 0.1805
St. M.	0.901 ± 0.0368	0.989 ± 0.0486	0.947 ±0.0519
	**0.951** ± 0.0364	0.936 ± 0.0435	0.925 ± 0.0580
St. P.	**0.952** ± 0.0229	0.938 ± 0.0257	0.929 ± 0.0337
	0.914 ± 0.0429	0.969 ± 0.0272	0.958± 0.0332
OUL.	0.685 ± 0.0114	0.691 ± 0.0146	0.688 ±0.0125
	**0.787** ± 0.0773	0.759 ± 0.1103	0.691 ± 0.1659
KDD	**0.391** ± 0.0176	0.386 ± 0.0127	**0.391** ± 0.0175
	0.601 ± 0.0245	0.603 ± 0.0213	0.601 ± 0.0248

**KbPIB** and **JPIB** improve precision and recall in a big margin over the baseline **NF** across multiple datasets. Bold values are the best scores across different methods.

Comparing AUC ROC in Table 2, **JPIB** outperforms **KbPIB** in 10 and achieves at least the same performance in 14 out of 19 datasets. Comparing precision and recall in Table 3, precision of **JPIB** outperforms **KbPIB** in 10 and achieves at least the same performance in 11 datasets; recall of **JPIB** outperforms **KbPIB** in 6 datasets and achieves at least the same performance in 7 datasets. Overall, **JPIB** outperforms **KbPIB**, suggesting that updating the privileged model with the classifier improves gradient boosting with sensitive information (**Q2**).

Intuitively, we expect the gains from our approach to be relative to the quality of the privileged information. When privileged information is highly discriminative, we expect greater gains from our approach and vice versa. For the standard benchmark and medical datasets (ref. Table 2), there is a correlation between the quality of the privileged information and the performance. We compare the performance only using the privileged features with **KbPIB** and **JPIB**, respectively. The Pearson correlation values of the AUC ROC are 0.237 (**KbPIB**) and 0.306 (**JPIB**). This helps explain the reason that **JPIB** outperforms **KbPIB** overall (**Q2**).

#### 4.3.1. Prior framework for privileged information

**Figure 2:**
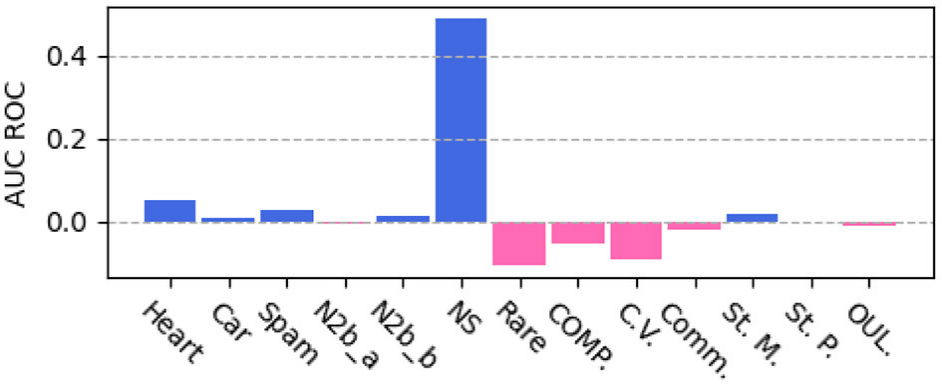
Performance comparison between JPIB and SVM+ (positive value if JPIB performs better; SVM+ fails to train on six large datasets).

To compare with previous study of using privileged information with SVM, we run SVM+ on our data splits and include results, as shown in Table 2. The major drawback of the previous study with SVM is that it lacks interpretability and cannot handle large datasets well. SVM+ runs slowly on large datasets (~20 k instances) and fails to train on datasets with ~30+k instances (6 datasets) due to out-of-memory error. As the performance difference between our method **JPIB** and SVM+, as shown in Figure 2, our method can outperform SVM+ (**Q1**). For some domains, our method **JPIB** gets lower AUC ROC compared with SVM+. This shows that SVM is still a very competitive base classifier. Applying our methods to a more powerful base classifier is a prospective future study to further improve the performance on more domains.

#### 4.3.2. Privileged information and fairness

We evaluate fairness on the fairness benchmark datasets (Table 4) and the real-world medical datasets. We compare against several fairness metrics: Statistical Parity (SP; Dwork et al., 2012), Equalized Odds (EO; Hardt et al., 2016), and Absolute Between-ROC Area (ABROCA; Gardner et al., 2019). SP measures the bias of predicting positive for different groups. We use SP to measure the overall fairness in predictive accuracy of our methods. EO measures the bias of predicting positive between different groups conditioned on the label. We take EO to further examine the fairness in predictive accuracy of our methods, specifically given different labels. ABROCA measures the divergence of ROC curves between different groups. ABROCA is adopted to quantify the fairness of our methods over all possible thresholds.


$$\begin{array}{l} SP=|P\left(ŷ=+|s=0\right)-P\left(ŷ=+|s=1\right)|\\ EO=\underset{v\in \left\{+,-\right\}}{\sum }|P\left(ŷ=+|s=1,y=v\right)-P\left(ŷ=+|s=0,y=v\right)|\\ ABROCA=\overset{1}{\underset{0}{\int }}|\text{RO}{\text{C}}_{1}\left(t\right)-\text{RO}{\text{C}}_{0}\left(t\right)|dt \end{array}$$

In addition to the previous baseline, we also compare against **All**, which learns a model that contains (imputed) privileged and classifier features. However, at test time, it estimates the privileged features based on the most common training value. Blue denotes when our approach outperforms **All** and bold denotes the best performance. As shown in Table 4, our approach achieves better fairness metrics than **All** in 10 (EO), 10 (SP), and 10 (ABROCA) datsests. Our approaches also perform at least as well as **NF** in 9 (EO), 9 (SP), and 13 (ABROCA) datasets. When considering the privacy or fairness of the resulting predictions, *imputing the privileged information by treating them as missing* has a clear **negative impact on the resulting fairness (see “All” in** **Table 4****)**. Collectively, our approaches are able to improve performance by leveraging sensitive privileged information while maintaining fairness (**Q3**).

**Table 4:** Scores of fairness metrics (lower values are better).

**Dataset**	**Metric**	**NF**	**KbPIB**	**JPIB**	**All**
N2b_b (race)	EO	0.112	0.071	**0.040**	0.075
	SP	0.017	0.020	**0.013**	0.023
	ABR.	0.115	0.114	**0.098**	0.129
Rare (mar.)	EO	**0.170**	0.337	0.360	0.526
	SP	**0.067**	0.126	0.130	0.155
	ABR.	0.249	**0.203**	0.246	0.268
Adult (sex)	EO	0.371	**0.314**	0.346	0.415
	SP	**0.049**	0.071	0.070	0.081
	ABR.	0.154	**0.130**	0.143	0.189
Diab. (sex)	EO	0.009	0.014	0.016	**0.007**
	SP	0.005	0.006	0.007	**0.003**
	ABR.	0.021	**0.019**	**0.019**	0.020
Dutch (sex)	EO	0.131	0.122	0.129	**0.110**
	SP	0.094	0.090	0.087	**0.066**
	ABR.	0.075	**0.058**	0.067	0.065
Bank (age)	EO	0.228	0.224	**0.178**	0.211
	SP	0.209	0.221	**0.183**	0.193
	ABR.	0.098	0.094	**0.088**	0.099
Credit (mar.)	EO	0.047	0.047	**0.037**	0.047
	SP	0.015	**0.013**	**0.013**	0.015
	ABR.	0.029	0.030	**0.025**	0.029
COMP. (race)	EO	0.247	0.239	**0.215**	0.334
	SP	0.146	0.141	**0.135**	0.200
	ABR.	0.071	0.059	0.061	**0.037**
C. V. (race)	EO	**0.190**	0.254	0.195	0.246
	SP	**0.089**	0.124	**0.089**	0.162
	ABR.	0.089	0.089	0.084	**0.059**
St. M. (sex)	EO	0.210	0.187	**0.184**	0.199
	SP	0.094	0.096	0.097	**0.089**
	ABR.	0.048	**0.038**	**0.038**	0.052
St. P. (sex)	EO	**0.320**	0.335	0.364	0.379
	SP	0.070	**0.069**	0.081	0.088
	ABR.	0.140	**0.124**	0.138	0.152
OUL. (sex)	EO	0.123	0.096	0.133	**0.082**
	SP	0.056	**0.039**	0.061	0.041
	ABR.	0.022	0.022	0.024	**0.021**
KDD (race)	EO	0.100	0.101	**0.099**	0.106
	SP	**0.054**	0.055	**0.054**	0.060
	ABR.	0.033	**0.032**	0.033	0.035

**KbPIB** and **JPIB** achieve significantly better fairness scores than the baseline **All** (sensitive features are imputed for test) and suppress the baseline **NF** over different metrics across multiple datasets. Bold values are the best scores across different methods.

**Figure 3:**
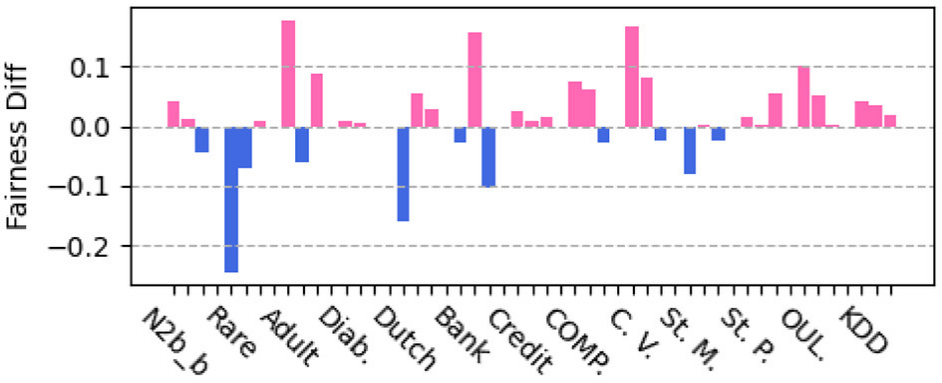
Fairness comparison between JPIB and MFC (negative value if JPIB is fairer).

To further verify the fairness benefit of our approach, we compare with MFC (Zafar et al., 2017). MFC learns fair classifiers by leveraging measurement of decision boundary (un)fairness, gaining fine-grained control on fairness with small cost of accuracy. As compared with MFC, our methods improve the prediction accuracy over the boosting baseline, and we would like to confirm that our methods enhance fairness. We apply MFC to our data splits and generate fairness scores on the same datasets of Table 4. Figure 3 shows the difference of scores of three fairness metrics between our approach **JPIB** and the baseline MFC on each dataset. We can observe that our approach **JPIB** achieves comparable fairness scores to MFC.

## 5. Conclusion

We considered the problem of learning with privileged and sensitive information using gradient boosting and proposed two algorithms that learned using these information. The extensive experiments in standard, medical, and fairness datasets demonstrated the ability of our algorithms to learn robust yet fair models. More extensive evaluation on large data sets, integration of other forms of domain knowledge into our framework, understanding the relationship with other fairness models, and considering more expressive models such as deep networks remain interesting future directions.

## Data availability statement

The original contributions presented in the study are included in the article/Supplementary material, further inquiries can be directed to the corresponding author.

## Ethics statement

The aim of our study is to use the sensitive features as privileged ones to avoid any discriminative social bias in our study. While we do not foresee many ethical issues with our study, it is conceivable that some sensitive features might be grouped under normal feature set. The risk for ethical issues when the privileged information is identified and flagged appropriately is low. The identification of sensitive features is the most important task and could potentially affect the results of the deployment of the algorithm. The code will be released publicly and maintained by the authors in GitHub.

## Author contributions

SY: Conceptualization, Data curation, Formal analysis, Investigation, Methodology, Software, Validation, Visualization, Writing—original draft, Writing—review & editing. PO: Investigation, Methodology, Writing—original draft, Writing—review & editing. RP: Conceptualization, Investigation, Writing—original draft. KK: Conceptualization, Investigation, Writing—original draft. SN: Conceptualization, Funding acquisition, Investigation, Methodology, Project administration, Supervision, Writing—original draft, Writing—review & editing.

## Footnotes

^1^We use $\psi \left(y_{i}=1,{\text{x}}_{i}^{\text{C}\text{F}}\right)$ to denote the probability mass of being positive given the classifier features.

^2^More details of derivation in Supplementary material.

^3^ https://github.com/starling-lab/PI_GBM

^4^ https://www.lalpathlabs.com/
