# Supplementary material for: Learning with privileged and sensitive information: a gradient-boosting approach
Source: Front Artif Intell. 2023 Nov 13;6:1260583. doi: 10.3389/frai.2023.1260583 (PMC10679679; doi:10.3389/frai.2023.1260583)
Supplement: Supplementary file 1 [file Data_Sheet_1.PDF]

Supplementary Material

1 DERIVATION OF OBJECTIVE

1.1 Objective

Given the data features X and class labels Y for n instances, the features and class label of the i-th instance is $\mathbf{x}_i$ and y_i. The likelihood of a model is

$$\prod_i P(y_i|\mathbf{x}_i;\psi)$$

Model parameters are represented by ψ.

For the ease of optimization and the monotonicity of logarithm, we consider log-likelihood. The log-likelihood (LL) objective is

$$\text{LL}(Y,X;\psi) = \sum_i \log P(y_i|\mathbf{x}_i;\psi) \tag{S1}$$

To maximize the log-likelihood, we get

$$\text{MLL}(Y,X;\psi) = \max_\psi \sum_i \log P(y_i|\mathbf{x}_i;\psi)$$

In general,

$$P(y|\mathbf{x};\psi) = \frac{\exp\big(\psi(y,\mathbf{x})\big)}{\sum_{y'}\exp\big(\psi(y',\mathbf{x})\big)} \tag{S2}$$

In order to leverage the privileged features which are not available during testing but available during training, we propose the objective

$$\sum_i \Big(\log P(y_i|\mathbf{x}_i^{\mathbf{CF}};\psi) - \alpha\cdot \text{KL}\big(P(y_i|\mathbf{x}_i^{\mathbf{PF}};\psi')||P(y_i|\mathbf{x}_i^{\mathbf{CF}};\psi)\big)\Big) \tag{S3}$$

$\mathbf{x}_i^{\mathbf{CF}}, \mathbf{x}_i^{\mathbf{PF}}$ represents the normal features and privileged features of the i-th instance. ψ represents parameters of the model on normal features. ψ' represents parameters of the model on privileged features. We find the model parameters ψ to not only maximize the log-likelihood of the model on normal features, but also minimize the distance between probability distributions built on privileged features and normal features. The model built on privileged features can provide richer information than mere class labels. Hence the model on normal features learned under this objective is supposed to perform better.

1.2 Gradient

For functional gradient boosting, we calculate derivative of Equation S1,

$$
\begin{aligned}
\frac{\partial \mathrm{LL}(Y,X;\psi)}{\partial \psi(y_i,\mathbf{x}_i)} &= \partial \sum_j \log \frac{\exp\big(\psi(y_j,\mathbf{x}_j)\big)}{\sum_{y'} \exp\big(\psi(y',\mathbf{x}_j)\big)} / \partial \psi(y_i,\mathbf{x}_i) \\
&= \partial \log \frac{\exp\big(\psi(y_i,\mathbf{x}_i)\big)}{\sum_{y'} \exp\big(\psi(y',\mathbf{x}_i)\big)} / \partial \psi(y_i,\mathbf{x}_i) \\
&= \frac{\partial\Big(\psi(y_i,\mathbf{x}_i) - \log \sum_{y'} \exp\big(\psi(y',\mathbf{x}_i)\big)\Big)}{\partial \psi(y_i,\mathbf{x}_i)} \\
&= 1 - \frac{\psi(y_i,\mathbf{x}_i)}{\sum_{y'} \exp\big(\psi(y',\mathbf{x}_i)\big)} \\
&= 1 - P(y_i|\mathbf{x}_i;\psi)
\end{aligned}
$$

For binary classification, the derivative with regard to the positive class $\psi(y_i = 1, \mathbf{x}_i)$ of Equation S1 is

$$
\begin{aligned}
&\frac{\partial \mathrm{LL}(Y,X;\psi)}{\partial \psi(y_i=1,\mathbf{x}_i)} \\
&= \frac{\partial \sum_j \log P(y_j|\mathbf{x}_j;\psi)}{\partial \psi(y_i=1,\mathbf{x}_i)} \\
&= \frac{\partial \log P(y_j|\mathbf{x}_j;\psi)}{\partial \psi(y_i=1,\mathbf{x}_i)} \\
&= \frac{\partial\Bigg(\psi(y_i,\mathbf{x}_i) - \log\Big(\exp\big(\psi(y_i=1,\mathbf{x}_i)\big) + \exp\big(\psi(y_i=0,\mathbf{x}_i)\big)\Big)\Bigg)}{\partial \psi(y_i=1,\mathbf{x}_i)} \\
&= I(y_i=1) - \frac{\exp\big(\psi(y_i=1,\mathbf{x}_i)\big)}{\exp\big(\psi(y_i=1,\mathbf{x}_i)\big) + \exp\big(\psi(y_i=0,\mathbf{x}_i)\big)} \\
&= I(y_i=1) - P(y_i=1|\mathbf{x}_i;\psi) \qquad \text{(S4)}
\end{aligned}
$$

To incorporate the guide for positive class from privileged features, we include additional KL divergence to the objective. The derivative of KL divergence to $\psi(y_i = 1, \mathbf{x}_i^{\mathbf{CF}})$,

$$
\begin{aligned}
&\frac{\partial \mathrm{KL}\big(P(y_i|\mathbf{x}_i^{\mathbf{PF}};\psi')||P(y_i|\mathbf{x}_i^{\mathbf{CF}};\psi)\big)}{\partial\psi(y_i=1,\mathbf{x}_i^{\mathbf{CF}})} \\
&= \frac{\partial \sum_{y_i} P(y_i|\mathbf{x}_i^{\mathbf{PF}};\psi')\big[\log P(y_i|\mathbf{x}_i^{\mathbf{PF}};\psi') - \log P(y_i|\mathbf{x}_i^{\mathbf{CF}};\psi)\big]}{\partial\psi(y_i=1,\mathbf{x}_i^{\mathbf{CF}})} \\
&= -\frac{\partial \sum_{y_i} P(y_i|\mathbf{x}_i^{\mathbf{PF}};\psi')\log P(y_i|\mathbf{x}_i^{\mathbf{CF}};\psi)}{\partial\psi(y_i=1,\mathbf{x}_i^{\mathbf{CF}})} \\
&= -\Big(\frac{\partial P(y_i=1|\mathbf{x}_i^{\mathbf{PF}};\psi')\log P(y_i=1|\mathbf{x}_i^{\mathbf{CF}};\psi)}{\partial\psi(y_i=1,\mathbf{x}_i^{\mathbf{CF}})} + \frac{\partial P(y_i=0|\mathbf{x}_i^{\mathbf{PF}};\psi')\log P(y_i=0|\mathbf{x}_i^{\mathbf{CF}};\psi)}{\partial\psi(y_i=1,\mathbf{x}_i^{\mathbf{CF}})}\Big) \\
&= -\big(P(y_i=1|\mathbf{x}_i^{\mathbf{PF}};\psi')\frac{\partial\log P(y_i=1|\mathbf{x}_i^{\mathbf{CF}};\psi)}{\psi(y_i=1,\mathbf{x}_i^{\mathbf{CF}})} + P(y_i=0|\mathbf{x}_i^{\mathbf{PF}};\psi')\frac{\partial\log P(y_i=0|\mathbf{x}_i^{\mathbf{CF}};\psi)}{\partial\psi(y_i=1,\mathbf{x}_i^{\mathbf{CF}})}\big)
\end{aligned}
$$

We substitute the derivatives by result from Equation S4,

$$
\begin{aligned}
&= -\Big(P(y_i=1|\mathbf{x}_i^{\mathbf{PF}};\psi')\cdot\big(1-P(y_i=1|\mathbf{x}_i^{\mathbf{CF}};\psi)\big)+ \\
&\qquad P(y_i=0|\mathbf{x}_i^{\mathbf{PF}};\psi')\cdot\big(-P(y_i=1|\mathbf{x}_i^{\mathbf{CF}};\psi)\big)\Big) \\
&= P(y_i=1|\mathbf{x}_i^{\mathbf{CF}};\psi) - P(y_i=1|\mathbf{x}_i^{\mathbf{PF}};\psi')
\end{aligned}
\tag{S5}
$$

Combining the result from Equation S4, we can get the derivative of the objective in Equation S3 with regard to $\psi(y_i = 1, \mathbf{x}_i^{\mathbf{CF}})$,

$$
\begin{aligned}
&I(y_i=1) - P(y_i=1|\mathbf{x}_i^{\mathbf{CF}};\psi) \\
&\qquad - \alpha\cdot\big(P(y_i=1|\mathbf{x}_i^{\mathbf{CF}};\psi) - P(y_i=1|\mathbf{x}_i^{\mathbf{PF}};\psi')\big)
\end{aligned}
\tag{S6}
$$

1.3 Hessian

To make the objectives work for XGBoost (Chen and Guestrin, 2016), we need to calculate the second-order derivative, hessian. From Equation S4, the second-order derivative of the log-likelihood is,

$$
\frac{\partial(I(y_i=1)-P(y_i=1|\mathbf{x}_i;\psi))}{\partial\psi(y_i=1,\mathbf{x}_i)} = -\frac{\partial P(y_i=1|\mathbf{x}_i;\psi)}{\psi(y_i=1,\mathbf{x}_i)}
$$

Substitute $P(y_i = 1|\mathbf{x}_i; \psi)$ by Equation S2,

$$= -\left[\frac{\exp\big(\psi(y_i = 1, \mathbf{x}_i)\big)}{\exp\big(\psi(y_i = 1, \mathbf{x}_i)\big) + \exp\big(\psi(y_i = 0, \mathbf{x}_i)\big)} - \left(\frac{\exp\big(\psi(y_i = 1, \mathbf{x}_i)\big)}{\exp\big(\psi(y_i = 1, \mathbf{x}_i)\big) + \exp\big(\psi(y_i = 0, \mathbf{x}_i)\big)}\right)^2\right]$$

$$= -\Big[P(y_i = 1|\mathbf{x}_i; \psi) - \big(P(y_i = 1|\mathbf{x}_i; \psi)\big)^2\Big]$$

$$= -P(y_i = 1|\mathbf{x}_i; \psi)\big(1 - P(y_i = 1|\mathbf{x}_i; \psi)\big) \tag{S7}$$

From Equation S5, the second-order derivative of KL divergence

$$\frac{\partial\big(P(y_i = 1|\mathbf{x}_i^{\mathbf{CF}}; \psi) - P(y_i = 1|\mathbf{x}_i^{\mathbf{PF}}; \psi')\big)}{\partial\psi(y_i = 1, \mathbf{x}_i^{\mathbf{CF}})}$$

$$= \frac{\partial P(y_i = 1|\mathbf{x}_i^{\mathbf{CF}}; \psi)}{\partial\psi(y_i = 1, \mathbf{x}_i^{\mathbf{CF}})}$$

Similar as hessian of log-likelihood in Equation S7

$$= P(y_i = 1|\mathbf{x}_i^{\mathbf{CF}}; \psi)\big(1 - P(y_i = 1|\mathbf{x}_i^{\mathbf{CF}}; \psi)\big) \tag{S8}$$

Combine the hessian of log-likelihood and KL divergence, the hessian of the new objective

$$- P(y_i = 1|\mathbf{x}_i^{\mathbf{CF}}; \psi)\big(1 - P(y_i = 1|\mathbf{x}_i^{\mathbf{CF}}; \psi)\big) - \alpha \cdot P(y_i = 1|\mathbf{x}_i^{\mathbf{CF}}; \psi)\big(1 - P(y_i = 1|\mathbf{x}_i^{\mathbf{CF}}; \psi)\big)$$

$$= -(1 + \alpha) \cdot \Big[P(y_i = 1|\mathbf{x}_i^{\mathbf{CF}}; \psi)\big(1 - P(y_i = 1|\mathbf{x}_i^{\mathbf{CF}}; \psi)\big)\Big]$$

Since XGBoost takes loss function as objective and its gradient and hessian, a negative sign needs to be added to gradient and hessian of our objective.

2 ALGORITHMS

Algorithm 1 describes the learning of the baseline $\mathbf{NF}$ model. Lines 2 to 8 follow the procedure of learning gradient boosted decision trees regarding the objective in Equation S1, with early-stopping strategy in Algorithm 2.

3 PARAMETERS

The values of α for $\mathbf{KbPIB}$ are Heart 0.016, Car 0.036, Spam 0.048, N2b_a 0.198, N2b_b 0.764, NS 0.001, Rare 0.615, Adult 0.936, Diab. 0.173, Dutch 0.719, Bank 0.158, Credit 0.035, COMP. 0.147, C. V. 0.584, Comm. 0.345, St. M. 0.065, St. P. 0.198, OUL. 0.42, KDD 0.067. The values of α for $\mathbf{JPIB}$ are Heart 0.018, Car 0.736, Spam 0.078, N2b_a 0.854, N2b_b 0.986, NS 0.001, Rare 0.653, Adult 0.01, Diab. 0.141, Dutch 0.213, Bank 0.07, Credit 0.239, COMP. 0.960, C. V. 0.379, Comm. 0.216, St. M. 0.066, St. P. 0.028, OUL. 0.013, KDD 0.039. Thresholds used for precision and recall can be found in source code files.

Algorithm 1 NF: Normal (Classifier) Features

Input: Training data X_{train}, Y_{train}; validation data X_{val}, Y_{val}
Parameter: Number of trees N, early-stop patience P
Output: Learned model ψ

Initialize model $\psi_0 = 0$, counter $C = 0$, score R, best number of trees index j
for $i = 1$ **to** N **do**
  $\Delta_i \leftarrow \text{ComputeGradient}(X_{\text{train}}, Y_{\text{train}}, \psi_{i-1})$ {Eq. S4}
  $\hat{\Delta}_i \leftarrow \text{FitRegressionValue}(X_{\text{train}}, \Delta_i)$
  $\psi_i \leftarrow \psi_{i-1} + \hat{\Delta}_i$
  $R_{\text{val}} \leftarrow \text{Evaluate}(X_{\text{val}}, Y_{\text{val}}, \psi_i)$
  j, R, $C \leftarrow$ EarlyStop(i, j, R, R_{val}, C, P) {Alg. 2}
end for
return ψ_j

Algorithm 2 EarlyStop

Input: i, j, R, R_{v}, C, P
Output: j, R, C

if $i = 1$ **then**
  $R \leftarrow R_{\text{v}}$, $j \leftarrow 1$
else if $R_{\text{v}} \leq R$ **then**
  $C \leftarrow C + 1$
else
  $C \leftarrow 0$, $R \leftarrow R_{\text{v}}$, $j \leftarrow i$
end if
if $C = P$ **then**
  break
end if
return j, R, C

4 RESULTS

The AUC ROC results with standard deviation of **NF**, **KbPIB**, **JPIB** and SVM+ are in Table S1.

REFERENCES

Chen, T. and Guestrin, C. (2016). Xgboost: A scalable tree boosting system. In *KDD*

Table S1. AUC ROC. **KbPIB** and **JPIB** outperform the baseline **NF** in nearly all the datasets. ”-” indicates out-of-memory error.

Dataset	NF	KbPIB	JPIB	SVM+
Heart	0.792 ±0.0754	**0.810** ±0.0776	0.798 ±0.0713	0.746 ±0.0788
Car	0.845 ±0.0284	**0.846** ±0.0276	**0.846** ±0.0283	0.841 ±0.0226
Spam	0.961 ±0.0076	0.961 ±0.0082	**0.962** ±0.0075	0.934 ±0.0109
N2b_a	0.658 ±0.0757	0.656 ±0.0699	0.684 ±0.0810	**0.690** ±0.0638
N2b_b	0.643 ±0.0486	0.652 ±0.0504	**0.655** ±0.0636	0.641 ±0.0777
NS	0.989 ±0.0248	0.989 ±0.0248	0.989 ±0.0248	0.5 ±0.0
Rare	0.531 ±0.2015	0.614 ±0.0954	0.560 ±0.1904	**0.667** ±0.1241
Adult	0.714 ±0.0202	**0.725** ±0.0484	0.719 ±0.0260	- -
Diab.	0.562 ±0.0061	0.561 ±0.0106	**0.566** ±0.0063	- -
Dutch	0.744 ±0.0319	0.763 ±0.0334	**0.764** ±0.0318	- -
Bank	0.681 ±0.0292	0.696 ±0.0408	**0.714** ±0.0230	- -
Credit	0.701 ±0.0122	**0.703** ±0.0163	**0.703** ±0.0175	- -
COMP.	0.618 ±0.0360	0.627 ±0.0343	0.643 ±0.0455	**0.698** ±0.0174
C. V.	0.567 ±0.0901	0.596 ±0.0669	0.609 ±0.0492	**0.703** ±0.0302
Comm.	0.893 ±0.0587	0.883 ±0.0588	0.899 ±0.0552	**0.919** ±0.0453
St. M.	0.959 ±0.0257	0.974 ±0.0204	**0.975** ±0.0202	0.959 ±0.0209
St. P.	0.908 ±0.0773	**0.921** ±0.0432	0.914 ±0.0521	0.914 ±0.0365
OUL.	0.523 ±0.0179	0.532 ±0.0235	0.523 ±0.0143	**0.534** ±0.0213
KDD	0.889 ±0.0046	**0.890** ±0.0042	**0.890** ±0.0046	- -
